# Using Virtual Reality in a Rehabilitation Program for Patients With Breast Cancer: Phenomenological Study

**DOI:** 10.2196/44025

**Published:** 2024-04-16

**Authors:** Shih-Chung Wu, Chia-Wen Chuang, Wen-Chun Liao, Chung-Fang Li, Hsin-Hsin Shih

**Affiliations:** 1 Department of Surgery, Kaohsiung Chang Gung Memorial Hospital, Kaohsiung, Taiwan; 2 Department of Nursing, Kaohsiung Chang Gung Memorial Hospital, Kaohsiung, Taiwan; 3 Department of Public Health, China Medical University, Taichung, Taiwan; 4 School of Nursing, College of Healthcare, China Medical University, Taichung, Taiwan; 5 Department of Nursing, China Medical University Hospital, Taichung, Taiwan

**Keywords:** breast cancer, rehabilitation, virtual reality, VR, virtual reality design process, VR design process, feasibility, accessibility

## Abstract

**Background:**

Surgery is an essential treatment for early-stage breast cancer. However, various side effects of breast cancer surgery, such as arm dysfunction and lymphedema, remain causes for concern. Rehabilitation exercises to prevent such side effects should be initiated within 24 hours after surgery. Virtual reality (VR) can assist the process of rehabilitation; however, the feasibility of applying VR for rehabilitation must be explored, in addition to experiences of this application.

**Objective:**

This study explored patients’ attitudes toward and experiences of using VR for their rehabilitation to determine the feasibility of such VR use and to identify potential barriers.

**Methods:**

A phenomenological qualitative study was conducted from September to December 2021. A total of 18 patients with breast cancer who had undergone surgical treatment were interviewed using open-ended questions. The Colaizzi 7-step procedure for phenomenological analysis was used for data analysis. To ensure high study reliability, this study followed previously reported quality criteria for trustworthiness.

**Results:**

Three themes were identified: (1) VR was powerful in facilitating rehabilitation, (2) early and repetitive upper limb movements were an advantage of VR rehabilitation, and (3) extensive VR use had challenges to be overcome. Most of the interviewed patients reported positive experiences of using VR for rehabilitation. Specifically, VR helped these patients identify appropriate motion and angle limits while exercising; in other words, knowledge gained through VR can play a key role in the rehabilitation process. In addition, the patients reported that the use of VR provided them company, similar to when a physiotherapist is present. Finally, the gamified nature of the VR system seemed to make VR-based rehabilitation more engaging than traditional rehabilitation, particularly with respect to early rehabilitation; however, the high cost of VR equipment made VR-based rehabilitation difficult to implement at home.

**Conclusions:**

The interviewed patients with breast cancer had positive experiences in using VR for rehabilitation. The high cost of both VR equipment and software development presents a challenge for applying VR-based rehabilitation.

## Introduction

Breast cancer is a major global health problem. In Taiwan, more than 10,000 women are diagnosed with breast cancer every year; approximately 80% of these diagnoses are early-stage breast cancer and most require surgical treatment [1]. More than 1 in 5 women who are breast cancer survivors might eventually develop upper limb lymphedema [2]. In addition, the life expectancy of patients with stage 0 to stage 3 breast cancer is 20 to 32 years [3]. Breast cancer treatments include surgery, radiation therapy, chemotherapy, endocrine therapy, and targeted therapy. Because of advancements in diagnosis and treatment in recent years, the 5-year relative survival rate of breast cancer is now higher than 80% [4]. However, both ancillary dissection and axillary radiation are known to increase the incidence of lymphedema and axillary web syndrome [5]; associated symptoms include pain, upper extremity weakness, paresthesia, and limited range of motion (ROM), each of which can not only delay the commencement of radiation therapy but also lead to patients being unable to perform basic self-care tasks. This is a problem because less-mobile patients are more likely to experience side effects after surgery; for example, a frozen shoulder is a side effect that commonly occurs in the short term as a result of immobility after surgery [6]. These associated symptoms may persist for up to 1.5 years after surgery and therefore predispose patients to depression and other mental health conditions that can become long-term health problems [7,8].

Studies have suggested that the early commencement of postoperative rehabilitation exercises, including upper extremity abduction and flexion exercises, can reduce the risk of upper extremity pain and dysfunction [9,10]. Although the optimal time to start ROM exercises remains unclear, one study suggested that activity recovery tends to be more successful following earlier rehabilitation [11]. Typically, rehabilitation is recommended to be commenced within the first 7 days after surgery, with shoulder ROM initially being limited to 90° [12]. Hence, a patient’s willingness to undergo rehabilitation after surgery to prevent their arms from becoming motionless is essential. However, after surgery, a patient’s motivation to undergo physical rehabilitation tends to be low because of wound pain and fear of movement [13,14].

Virtual reality (VR) can reduce fear of movement and improve motivation to engage in rehabilitation. In addition, VR can promote compliance and rehabilitation success among patients [15-17]. Furthermore, VR appears to be effective in increasing shoulder ROM compared with standard physiotherapy for postoperative rehabilitation in patients with breast cancer [18]. The use of VR for rehabilitation is not new; however, feasibility studies that use VR to provide individualized, progressive practice for arm movements after surgery for breast cancer remain scarce. Feasibility research is often conducted prior to a randomized controlled trial [19]. In recent years, most studies investigating VR for rehabilitation in patients with breast cancer have used a randomized controlled design and have been prospective, adequately powered, and methodologically rigorous. Nevertheless, according to According to the recommendations of the VR-CORE (Clinical Outcomes Research Experts) [20], the development of new VR content should include 3 phases: VR1 to VR3. VR1 provides guidance for the development of new VR content; VR2 constitutes early testing related to the feasibility, acceptability, tolerability, and initial clinical efficacy of VR; and VR3 evaluates the efficacy of VR in comparison with controls. Bypassing feasibility and accessibility tests to jump immediately to clinical trials may lead to a gap between program development and bedside application and cause barriers to implementing VR in practice. Even though there is sound evidence that VR is effective in improving rehabilitation effects in patients with breast cancer, VR is not currently used in many clinical settings. According to Brennan et al [21], barriers to the implementation of VR include usability problems, cognitive limitations, cost, a lack of patient safety, low patient motivation, and negative patient responses. In addition, VR’s unfamiliarity to many patients may dampen their willingness to use VR [22]; thus, patients’ attitudes, experiences, and opinions related to VR must be understood. Further explorations of feasibility and accessibility are recommended for the use of VR in health care [23,24].

To the best of our knowledge, most studies regarding postoperative rehabilitation in patients with breast cancer have been clinical trials (ie, VR3) that evaluated only treatment effects, such as pain, grip power, muscle power, or ROM; VR2 studies are skipped. This study used a phenomenological approach to investigate the feasibility and experiences of a VR rehabilitation program (ie, this was a VR2 study) in patients with breast cancer. Therefore, this study was conducted to answer the following questions: (1) What are the lived experiences of patients using VR for arm rehabilitation? (2) How do they perceive the process? In answering these questions, this study determined the clinical effectiveness of and barriers to VR rehabilitation as a prelude to a definitive randomized controlled trial.

## Methods

### Design and Development of the VR System

**Figure 1.**
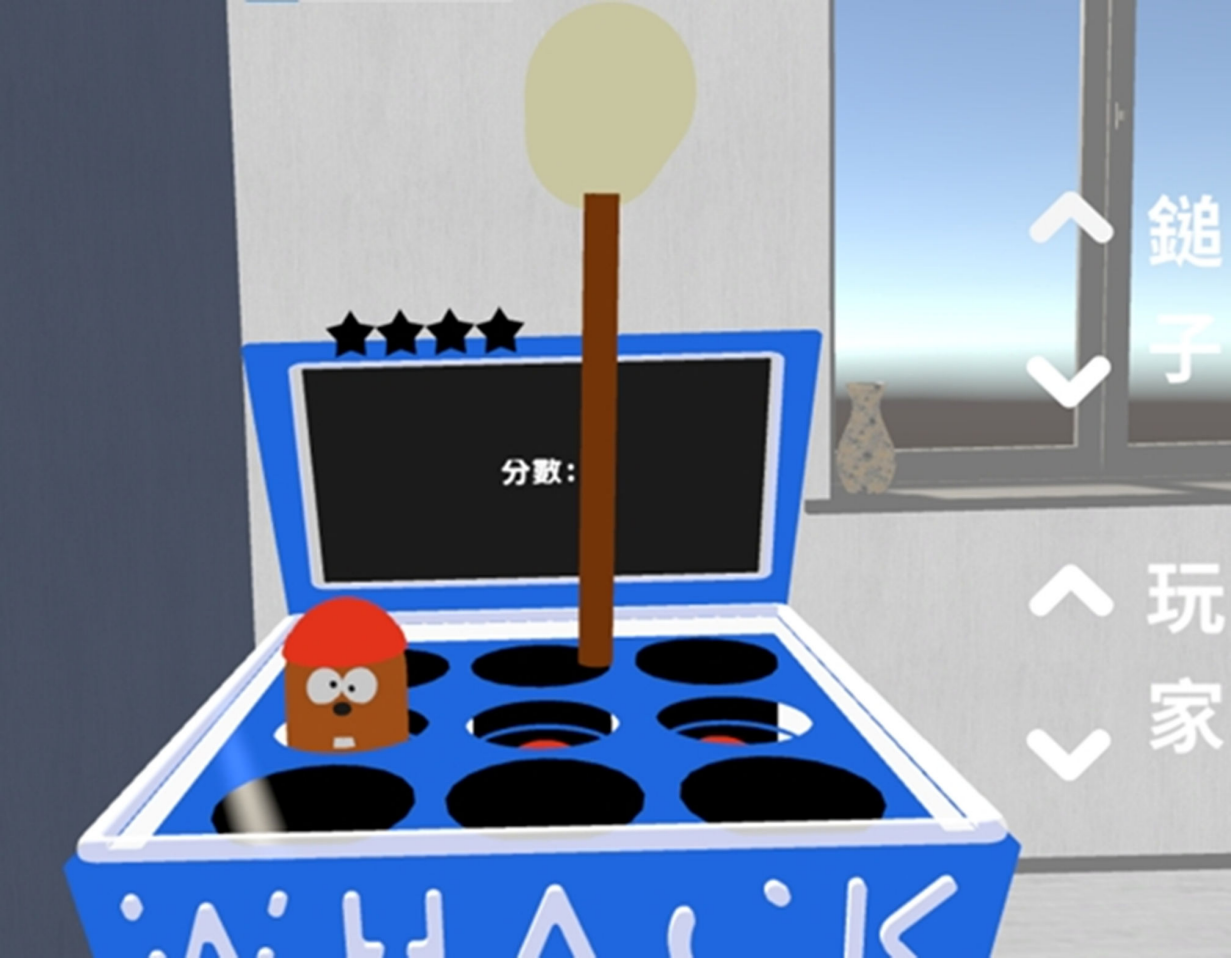
Whac-A-Mole–like scenario.

**Figure 2.**
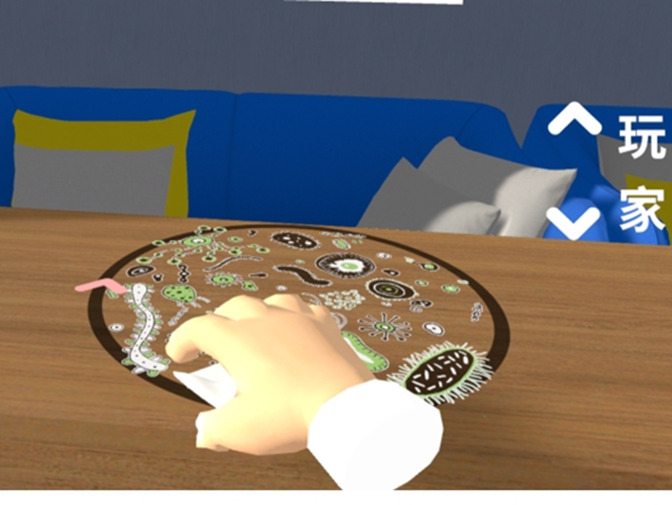
Wiping a table scenario.

**Figure 3.**
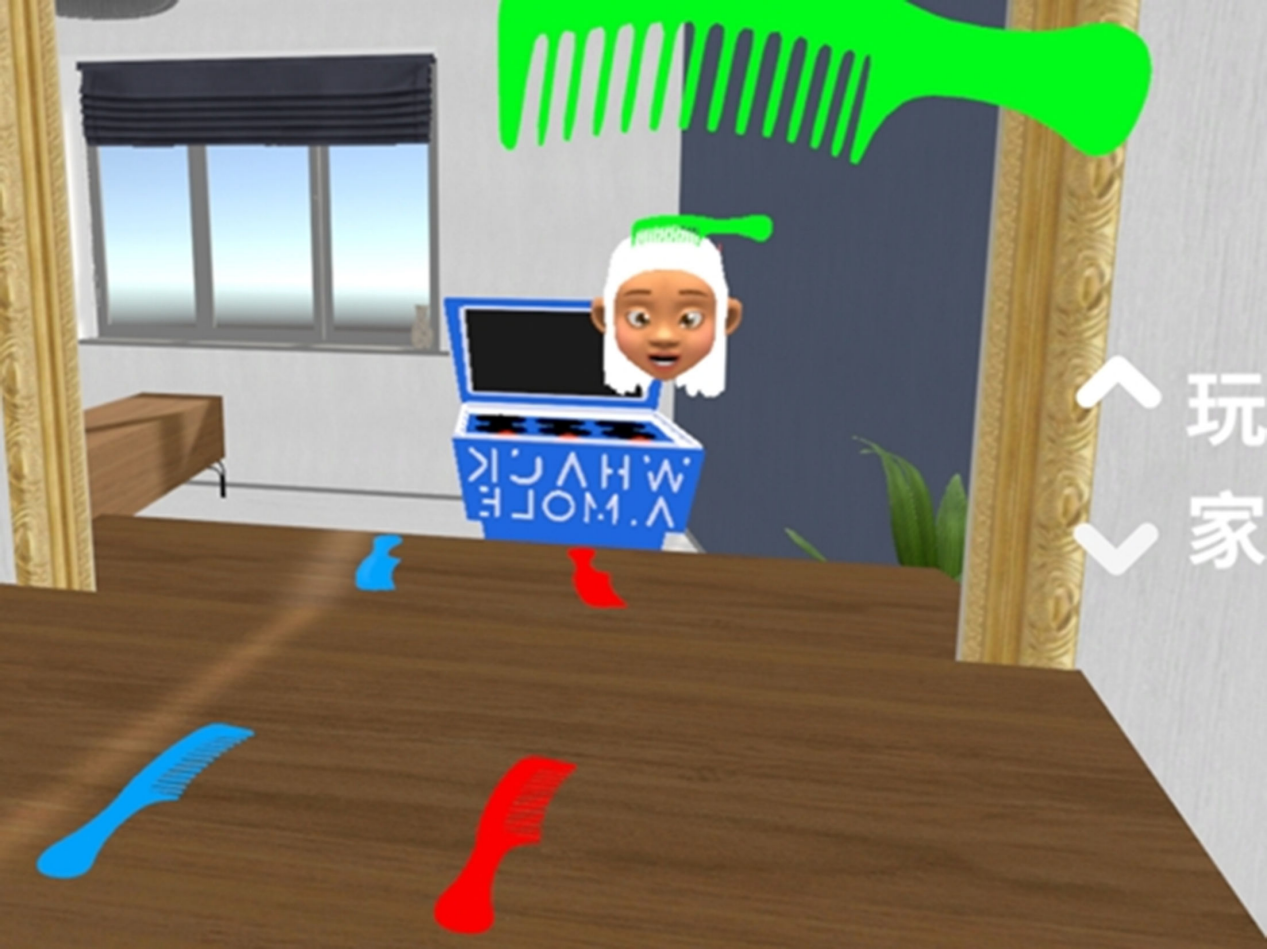
Combing one’s hair scenario.

The VR system in this study was designed and developed in accordance with the VR–CORE–VR1 guidelines [20]. Our VR design team was a multidisciplinary team comprising users and technicians. The rehabilitation program was developed in 3 stages. The first stage was based on inspiration gained through empathy. Specifically, interviews were held with 2 patients who were invited to share their rehabilitation experiences and express their perspectives and opinions to facilitate the design of an effective VR-based treatment program. The interview questions in this stage considered (1) smart device use behavior and (2) willingness and acceptability to use VR rehabilitation systems. The second stage involved ideation through team collaboration. Specifically, our team comprised experts in surgery, rehabilitative physicians, and software engineering, as well as a case manager. Innovative ideas were generated through methods such as brainstorming and collective ideation, and the ideas that were most suitable for prototyping were then compiled. The third stage involved prototyping based on user feedback. Specifically, further discussions were held with the aforementioned 2 patients to obtain further information. Our team then built prototypes to test 3 VR rehabilitation exercises prior to implementation (Table 1). Ultimately, we extracted 3 key sets of limb motions that are described as follows. The first motion was a Whac-A-Mole–like game that involved the abduction of the shoulder (no more than 90°). The second motion was wiping a table and involved external forearm rotation. The third motion was combing one’s hair and involved flexion of the shoulder (no more than 90°) (Figures 1-3). This study used the PC-based Oculus Quest 2 head-mounted display (HMD) to provide the most immersive VR experience possible.

**Table 1.** Summary of our design principles and strategies based on recommendations for best practices in VR1 (virtual reality phase 1) studies [20].

Design principles and strategies	Our practices
Inspiration through empathy, recruitment, observation, patient interviews, and expert interviews	Two patients with breast cancer who had experienced axillary web syndrome were invited for individual interviews regarding their relevant needs, experiences, fears, and expectations; a group discussion was then conducted to determine the patients’ needs.
Ideation through team collaboration, sharing stories and notes, and generating ideas	We analyzed, aggregated, and discussed the stories and data obtained in the previous phase. Our team then formulated 10 ideas for rehabilitation actions. After considering the restrictions on drainage tube indwelling the day after surgery (shoulder joint movement must not exceed 90°), we extracted 3 motions: (1) Whac-A-Mole, which involved abduction of the shoulder; (2) wiping a table, which involved external forearm rotation; and (3) combing one’s hair, which involved flexion of the shoulder.
Prototyping through continuous feedback, building a prototype, and repeatedly testing the prototype	We drew the height of a mallet on a wall and placed a picture of a gopher on a table to simulate the Whac-A-Mole game. For the wiping the table and combing hair scenarios, we used a table and a comb, respectively, and we then collected feedback from the patients after their first use. Our team found that the Whac-A-Mole game was difficult to perform owing to the different heights of the patients and seats (eg, a patient may need to raise their shoulder angle excessively to pick up the mallet, leading to pain and an inability to perform the exercise, forcing it to be abandoned). Thus, the software engineer adjusted the height of the controllable mallet and the adjustable gopher table to meet the need of each individual patient.

### Study Design

This qualitative study was guided by the phenomenological methodology; it focused on investigating the perceived experience of using VR rehabilitation and then used an inductive approach and the Colaizzi [25] interview data analysis method to identify themes from the VR rehabilitation experiences.

### Ethics Approval

Ethical approval for this study was granted by the Human Trials Ethics Committee of Chang Gung Memorial Hospital, Taiwan (202001617A3C501). All the participants provided written informed consent to participate in this study.

### Setting

This study was conducted at a medical center in Taiwan that receives up to 20 new patients with breast cancer per month.

### Participants and Recruitment

The criteria for participation in this study were as follows: (1) a new diagnosis of breast cancer and being hospitalized for surgical treatment; (2) being aged 20 to 65 years; (3) being able to read and speak Mandarin Chinese or Taiwanese; (4) being willing to use a wearable VR HMD and having sufficient cognitive ability to understand and follow instructions, as well as to interact with the VR environment; (5) having sufficient physical ability (self-reported) to use the VR equipment in the VR rehabilitation program; and (6) being willing to be interviewed. After surgical treatment for breast cancer, patients were invited to a rehabilitation consultation to ask about their willingness to try VR rehabilitation and were recruited to participate in this study. The recruitment period ran from September to December 2021, with 18 patients interviewed.

### Data Collection

First, the participants filled out a questionnaires on demographic data (including age, gender, and type of breast surgery), the Distress Thermometer (DT) questionnaire, and the Chinese Health Questionnaire–12 (CHQ-12). The DT is a visual analog scale for routine screening for distress in all patients with cancer; scores range from 0 (indicating no distress) to 10 (indicating extreme distress). A cutoff score of ≥7 is optimal for distress in patients with a new diagnosis [26]. The CHQ-12 was modified from the General Health Questionnaire–12, a widely used psychometric test for screening mental problems, with a score of ≥4 indicating psychological distress [27].

After completing the questionnaires, a face-to-face interview was conducted with each participant for approximately 30 minutes. Interview guides were developed by the research team, which included an expert in qualitative studies and a clinical nurse specialist in breast cancer. After the content of the first interview was analyzed, the interview questions were discussed and modified with the research team.

During the interview process, the researchers actively listened and continuously clarified any perspectives and meanings that the participants wanted to express. Participants were allowed to freely express their views. All interviews were audiorecorded. After the data analysis, the researchers reviewed the data with the patients to ensure data accuracy and completeness.

### Inductive Content Analysis

According to Kyngäs [28], an inductive approach, which is common in qualitative research, should be used when no prior research has addressed a specific phenomenon or if existing knowledge is fragmented. Because prior studies had not investigated the experiences of patients with breast cancer to evaluate the effectiveness of VR-based rehabilitation, our study used an inductive approach to content analysis, which involved data reduction, data grouping, and, finally, concept formation. The content was analyzed with the Colaizzi [25] phenomenological analysis method, which involves a 7-step procedure for content analysis. Specifically, 3 researchers analyzed the data and grouped them into themes to enhance comprehension. First, the interview transcript was read to obtain an idea of its overall meaning. Second, a researcher extracted significant statements relevant to the purpose of the study. Third, each statement considered meaningful was recorded for accurate representation of the research data. Fourth, the researcher classified significant statements into categories based on their meaning. Fifth, the researcher grouped similar categories together and organized them into themes. Sixth, all themes presented were combined to produce an exhaustive description. Seventh, the results were presented to each participant for feedback and to verify their accuracy.

### Trustworthiness

To enhance the trustworthiness of our study, we referred to the Guba and Lincoln [29] quality criteria of credibility, transferability, dependability, and confirmability.

To establish confidence in the credibility of the findings, triangulation was used to examine the consistency of the data. Researchers presented their summaries of the categories and themes from data analysis and discussed any uncertainty about any category or theme by replaying and reanalyzing the interview recording.

High transferability was ensured through the interview process, including the stability of observations and the interrater reliability. As an interviewer, the researcher was attentive to the interviewees’ experiences and avoided bias arising from their values and beliefs. The interviewer was conscious about avoiding misperceptions about what the interviewees were saying to ensure the stability of observations. There was only 1 interviewer, hence interrater reliability was not measured.

The study had high dependability, as the data collecting procedures were approved by an expert in qualitative methodology, and the themes and subthemes that emerged were consistent among all researchers after the coding and recoding procedure during the data analysis.

Finally, during the data analysis stage, 3 researchers who were not part of the study team confirmed the collected data by distinguishing between the units of the main themes and subthemes to verify the main themes’ theoretical saturation and thus achieved confirmability.

## Results

### Characteristics of Participants

Table 2 shows the participants’ characteristics. The mean age of the participants was 46.66 (SD 8.05) years; 17 participants were female (95%) and 1 was male (5%); 11 were married (61%); 11 (61%) were full-time workers; and 7 (39%) were housekeepers or had no formal employment. Regarding the types of surgery received, 9 participants (50%) underwent mastectomy and sentinel lymph node biopsy, 4 (23%) underwent mastectomy and axillary lymph node dissection, 2 (11%) underwent mastectomy and reconstruction, and 3 (16%) underwent modified radical mastectomy. All participants had DT scores less than 7, indicating no distress. In the CHQ-12 items, some participants reported having shaking or numbness of limbs (n=3, 17%), losing a great deal of sleep because of worry (n=4, 22%), and losing confidence in themselves (n=4, 22%) (items 4, 5, and 8). However, all participants had CHQ-12 scores less than 4, indicating no mental problems. Finally, 7 (39%) and 11 (61%) of the participants reported moderate (4-6) and mild (1-3) wound pain on the Numeric Rating Scale for Pain, respectively.

**Table 2.** Distribution of sociodemographic variables of participants (N=18).

Variables		Participants, n (%)
**Sex**		
	Female	17 (95)
	Male	1 (5)
**Age group (years)**		
	35-44	6 (33)
	45-54	8 (44)
	55-64	4 (3)
**Educational level**		
	Senior high school	9 (50)
	College	9 (50)
**Distress**		
	Yes (DT^a^ score ≥7)	0 (0)
	No (DT score <7)	10 (100)
**Mental problems**		
	Yes (CHQ-12^b^ score ≥4)	0 (0)
	No (CHQ-12 score <4)	18 (100)
**Numerical Rating Scale for Pain score**		
	1-3	11 (61)
	4-6	7 (39)
**Marital status**		
	Married or in a domestic partnership	11 (61)
	Single	5 (28)
	Separated or divorced	2 (11)
**Employment status**		
	Employed	11 (61)
	Unable to work	7 (39)
**Type of breast surgery**		
	Mastectomy with sentinel lymph node biopsy	9 (50)
	Mastectomy with axillary lymph node dissection	4 (23)
	Mastectomy with reconstruction	2 (11)
	Modified radical mastectomy	3 (16)

a DT: Distress Thermometer.

b CHQ-12: Chinese Health Questionnaire–12.

### Themes

The following 3 themes were inductively extracted from the interview data: (1) VR was powerful in facilitating rehabilitation, (2) early and repetitive upper limb movements were an advantage of VR rehabilitation, and (3) extensive VR use had challenges to be overcome. Tables 3-5 present these themes in greater detail alongside their subthemes and interviewee statements.

**Table 3.** Subthemes and interviewee quotes related to theme 1: virtual reality (VR) was powerful in facilitating rehabilitation.

Subthemes	Quotes
Obtaining knowledge	“It turns out that some actions are unsuitable [because of the drainage tube]; after use, I have a better idea of the angle of movement” (participant 2). “After practicing with this system [VR] and being discharged from the hospital, I know that some actions are not suitable for me to do, so I will not do them randomly” (participant 3). “I was anxious, thinking that not moving my arms was suitable for wound healing” (participant 13). “I can adjust my range of motion according to my tolerance, and I will not feel frustrated” (participant 17).
Companionship	“This way of rehabilitation [VR] is like a kind of support for me when exercising because thephysiotherapist does not always accompany me” (participant 9). “The usage process is very simple. I think it is well executed in the hospital and I can concentrate on practicing” (participant 5). “The physiotherapist was very busy and left after teaching me the rehabilitation movement, so I had to do it independently” (participant 11). “I didn’t understand what this was at first, and I was a little scared, but it turned out that I was doing rehabilitation while playing games” (participant 1).

**Table 4.** Subthemes and interviewee quotes related to theme 2: early and repetitive upper limb movements are an advantage of virtual reality (VR) rehabilitation.

Subthemes	Quotes
Reduced fear of movement	“The VR rehab game method is more fun than the traditional rehab method because it lets me know I need to do rehab exercises; I think I can do it a few more times” (participant 1). “My time in the hospital was short, and it was a pity that I didn’t do VR many times” (participant 6). “I experienced no pain or discomfort during the VR rehab; I want to do it a few more times” (participant 7). “After playing Whac-A-Mole, I attempted to move my arm” (participant 11). “While using it, I temporarily forgot that I had surgery” (participant 14). “Initially, I was terrified of moving my arms, but now, I’m having so much fun that I don’t realize I’m doing rehab” (participant 18).
Enhanced motivation to engage in rehabilitation	“The VR program can be performed as soon as possible; I have been performing VR for seven days since surgery. In the execution of the Whac-A-Mole and wiping the table exercises, I did not have too much discomfort or pain, and I am doing better and better” (participant 15). “Although there is no VR at home, I can continue to do the VR movements, and I will not worry about my arm’s limited range of motion in the future” (participant 16). “After using VR for rehabilitation, I found that my condition was not as bad as I thought” (participant 7).

**Table 5.** Subthemes and interviewee quotes related to theme 3: extensive virtual reality (VR) use has challenges to be overcome.

Subthemes	Quotes
Safety of the real-world environment	“I was initially scared because I had never used VR” (participant 4). “Wearing a VR HMD, I can’t see the outside world, so I’m worried about hitting something” (participant 5).
Cost of virtual reality rehabilitation equipment is too high to extend to home use	“VR rehabilitation equipment is too expensive to buy and can only be used in hospitals” (participant 5). “Without equipment, there is no way to use VR for rehabilitation” (participant 8). “VR rehabilitation can only be used in hospitals; at home, we use traditional rehabilitation methods” (participant 17). “I hope to rent VR equipment for my home so I can use it anytime” (participant 18). “Although there is the Switch, finding a suitable sports game for rehabilitation is difficult” (participant 9).
Motion sickness	“After wiping the table, I felt a little dizzy” (participant 10). “My eyes constantly roll when wiping the table, which is a little uncomfortable” (participant 9).

### VR Was Powerful in Facilitating Rehabilitation

#### Overview

A key concept of this inductive theme was the “power” obtained from knowledge and companionship. Related subthemes and interviewee statements are presented in Table 3.

#### Obtaining Knowledge

A total of 12 (70%) of the participants described how the VR content helped them learn the most helpful rehabilitation movements and the angular limitations of their exercises and thus enabled them to perform these exercises safely and relatively early in the rehabilitation process.

#### Companionship

Most of the participants stated that playing games enabled them to feel as though they were interacting with health care professionals and experiencing companionship and support.

### Early and Repetitive Upper Limb Movements: An Advantage of VR Rehabilitation

#### Overview

After surgical treatment for breast cancer, rehabilitative exercises must be performed early. In this study, wound pain and discomfort during such exercises were the main concerns of the participants, leading to fear of movement. Related subthemes and interviewee statements are presented in Table 4.

#### Reduced Fear of Movement

A total of 14 (80%) of the participants stated that the gamified design of VR rehabilitation made it more interesting than traditional rehabilitation, while 7 of the patients experienced moderate pain after surgery. However, during the use of VR for rehabilitation, these patients reported no wound pain or discomfort. In addition, their willingness to engage in rehabilitation was enhanced by the second day after surgery.

#### Enhanced Motivation to Engage in Rehabilitation

Most of the participants described an immersive virtual environment that facilitated the performance of early rehabilitative exercises. In addition, the participants were subsequently able to continue performing the aforementioned 3 rehabilitation movements at home without VR.

### Extensive VR Use Has Challenges to Be Overcome

#### Overview

Most of the patients had safety concerns, mainly because while wearing the VR HMD, they were unable to pay attention to the environment around them. Consequently, some of the patients initially felt apprehensive, fearing collisions with nearby objects. In addition, high equipment costs limited the accessibility of the equipment for home use, and many of the patients expressed disappointment that they were able to use the system only in hospital. Related subthemes and interviewee statements are presented in Table 5.

#### Safety of the Real-World Environment

While the patients were wearing the VR HMD and engaging in VR-based exercise, they were unable to pay attention to the environment around them. Thus, patients had the not-unfounded fear that they might collide with nearby objects and were initially apprehensive. Therefore, patients should be in a clutter-free environment when engaging in VR rehabilitation.

#### Cost of VR Rehabilitation Equipment Is Too High to Extend to Home Use

The cost of VR equipment for rehabilitation is high, making it relatively inaccessible for home use. Each new technology faces the problem of high equipment cost in its initial stage, and similarly, the main problem for most of the participants in this study was that they wanted to continue using the rehabilitation equipment at home. They indicated that they thought it was a pity that the system could be used only in hospital.

#### Motion Sickness

We found that 3 of the participants reported the side effect of motion sickness after performing the wiping table exercise.

## Discussion

### Principal Findings

To the best of our knowledge, this study is the first phenomenological study to investigate the feasibility of an immersive VR experience for facilitating postoperative rehabilitation in patients with breast cancer. Through inductive thematic analysis, our study found VR to be an effective and suitable intervention for our patient population. Tables3  4 address feasibility in relation to 2 of the 3 inductive themes of this study: “VR was powerful in facilitating rehabilitation” and “early and repetitive upper limb movements are an advantage of VR rehabilitation.” Table 5 reveals the key finding that the high costs of VR hardware and software limit its at-home use. These results further support the recommendations of the VR CORE group [20]. However, the high cost of VR equipment remains a concern.

### VR Was Powerful in Facilitating Rehabilitation

This study found that “power” was an essential concept and that power was cultivated through knowledge and companionship. One key finding related to this inductive theme was the knowledge obtained by the individual. In our study, most of the participants were skeptical about their early postoperative rehabilitation, and some had no idea how to proceed with it; however, through VR, these patients gained information about rehabilitation, which then led to behavioral changes. A previous study noted the importance of people’s need to know which approach they should adopt to improve their health [30]. In addition, most patients with breast cancer desire information concerning recovery details; in particular, they want real-time, transparent information regarding appropriate rehabilitative measures [21]. Our findings were consistent with those of the 2 aforementioned studies. Another present finding was related to companionship; specifically, the participants reported that they felt as if a medical care team was with them in their rehabilitation in the VR world. This finding is notable because not every patient with breast cancer receives a rehabilitation exercise consultation after surgery. Our interactive VR system provides clear information regarding the commencement and completion of rehabilitation exercises, and its prompts align with how the clinical rehabilitation program operates (eg, 20 prompts for each motion). The literature notes that an ideal user experience must include interaction with VR applications in order to encourage and support users to complete clinical activities; this assertion is consistent with our findings [31].

### Early and Repetitive Upper Limb Movements: Advantage of VR Rehabilitation

VR elicited engagement in early postoperative rehabilitation in most of this study’s patients, seemingly stemming from their reduced fear of movement and their enhanced motivation. Early postoperative rehabilitation in patients who have undergone breast cancer surgery aims to improve shoulder mobility, arm mobility, or both. VR is believed to be as effective as conventional physiotherapy in improving upper limb function to facilitate the maintenance of activities of daily living [32]. Some of the participants in this study were initially apprehensive to undertake postoperative rehabilitation primarily because of fear related to insufficient knowledge of the appropriate degree of movement. Nevertheless, our VR rehabilitation system was based on the concept of gamification; the participants who used the system were attracted to the game screen, and consequently, their negative feelings—including fear, pain, and discomfort—were temporarily alleviated. In this manner, the VR system considerably enhanced the participants’ motivation to engage in rehabilitation; this finding was consistent with those of previous studies [18,33]. In addition, the game design can be adjusted according to the needs of the patient to prevent patients from giving up on movements that are either difficult or so simple that the desired training outcomes cannot be achieved by performing them.

### Challenges of Extensive VR Use to Be Overcome

The participants in this study were required to wear HMDs; thus, they could not see their surroundings during rehabilitation. Related studies that have recommended VR have stated that when using such technology, a space of at least 70 cm × 70 cm is required; however, in our study, the participants took a seated position to minimize the risk of tripping and thus needed only to pay attention to whether the seat back was affecting exercise safety. Although research has shown that the cost of HMDs has fallen in recent years [15], VR devices remain unaffordable for many people in Taiwan. Therefore, even if patients with breast cancer are interested in using this emerging technology for rehabilitation, doing so without an HMD is difficult. In addition, the development and maintenance costs of VR software and hardware are high, and thus, the cost of VR equipment remains a challenge for large-scale implementation in postoperative rehabilitation.

Motion sickness (MS) is a common physiological response to VR immersion [34]. Previous studies have shown that behavioral and dietary strategies and physical therapy, such as listening to music or chewing gum [35], are effective in alleviating MS symptoms. Although each movement in our VR rehabilitation system was accompanied by music with a brisk tempo, 3 of the patients in this study still experienced MS due to unsuitable seat height. A previous study demonstrated that positive emotions can relieve MS [36]; we found that most patients have positive emotions while using VR for rehabilitation. Accordingly, this paper suggests that the use of VR should be combined with emotional assessments to effectively prevent MS. With regard to feasibility, we found that the present VR system met the patients’ needs, conformed to their values, and satisfied their expectations. The system also provided an engaging and interactive rehabilitation environment that stimulated the patients’ motivation to engage in early and continuous rehabilitation and, in turn, reduced their risk of developing defensive functional impairments. Nevertheless, the accessibility of VR proved to be a challenge because of the difficulty of using VR technology at home. Thus, such interventions may tend to be limited to in-hospital use because not every patient can afford to purchase VR equipment and software.

### Study Limitations

This study has some limitations. First, we did not conduct a pilot study of the interview guide. However, after the content of the first interview was analyzed, the interview questions were discussed and modified with the research team to identify and address any potential issues in the data collection methods. It may be recommended to incorporate a pilot study in future research to ensure validity and robustness. Second, the development of VR technology to facilitate postoperative rehabilitation in patients with breast cancer is still in its infancy. In this study, only 3 rehabilitation exercises were developed and clinically tested. The facilitators of and barriers to rehabilitation were investigated. To determine the clinical efficacy of the proposed VR system, a large-scale randomized controlled trial is needed.

### Conclusion

Feasibility research related to health care is crucial for generating novel ideas and improvements to provide more effective care for patients. From the perspective of this study, rehabilitation is an iterative, active, and educational process of problem-solving that must focus on a patient’s behavior. VR technology was effective in facilitating rehabilitation by providing knowledge and companionship to participants. It helped reduce fear of movement and enabled early and repetitive upper limb movements. However, safety concerns, high equipment costs, and potential side effects like MS were reported as challenges to extensive VR use.

## Abbreviations

CHQ-12: Chinese Health Questionnaire–12
CORE: Clinical Outcomes Research Experts
DT: Distress Thermometer
HMD: head-mounted display
MS: motion sickness
ROM: range of motion
VR: virtual reality
